# Th17 CD4+ T-Cell as a Preferential Target for HIV Reservoirs

**DOI:** 10.3389/fimmu.2022.822576

**Published:** 2022-02-07

**Authors:** Constance Renault, Nicolas Veyrenche, Franck Mennechet, Anne-Sophie Bedin, Jean-Pierre Routy, Philippe Van de Perre, Jacques Reynes, Edouard Tuaillon

**Affiliations:** ^1^ Pathogenesis and Control of Chronic and Emerging Infections, INSERM U1058, University of Montpellier, Etablissement Français du Sang, Antilles University, Montpellier, France; ^2^ Virology Laboratory, CHU de Montpellier, Montpellier, France; ^3^ Chronic Viral Illness Service and Research Institute and Division of Hematology, McGill University Health Centre, Montreal, QC, Canada; ^4^ IRD UMI 233, INSERM U1175, University of Montpellier, Montpellier, France; ^5^ Infectious Diseases Department, CHU de Montpellier, Montpellier, France

**Keywords:** T-helper 17 cells, HIV infections, HIV reservoir, mucosal immunology, CD4-positive T cells, lymphocytes

## Abstract

Among CD4+ T-cells, T helper 17 (Th17) cells play a sentinel role in the defense against bacterial/fungal pathogens at mucosal barriers. However, Th17 cells are also highly susceptible to HIV-1 infection and are rapidly depleted from gut mucosal sites, causing an imbalance of the Th17/Treg ratio and impairing cytokines production. Consequently, damage to the gut mucosal barrier leads to an enhanced microbial translocation and systemic inflammation, a hallmark of HIV-1 disease progression. Th17 cells’ expression of mucosal homing receptors (CCR6 and α4β7), as well as HIV receptors and co-receptors (CD4, α4β7, CCR5, and CXCR4), contributes to susceptibility to HIV infection. The up-regulation of numerous intracellular factors facilitating HIV production, alongside the downregulation of factors inhibiting HIV, helps to explain the frequency of HIV DNA within Th17 cells. Th17 cells harbor long-lived viral reservoirs in people living with HIV (PLWH) receiving antiretroviral therapy (ART). Moreover, cell longevity and the proliferation of a fraction of Th17 CD4 T cells allow HIV reservoirs to be maintained in ART patients.

## Introduction

### Toward the Progressive Definition of Th17-Oriented CD4+ T Cells

Over the past decade, functional and phenotype characterizations of T helper (Th) cells have allowed the subsets of lymphocytes to be defined. Th cells’ subpopulations were initially identified among effector cells through their cytokine secretion profiles, eliciting functional characteristics for each type. Hence, subpopulations of effector CD4+ T lymphocytes are referred to as Th1, Th2, Th9, Th17, Th22, T follicular helper cells (Tfh), and Treg regulatory profiles.

The Th17 profile was first described in 2005 by Harrington et al. (1) from a subpopulation previously included in the Th1 profile. Th17 effector cells are characterized by interleukin 17 secretion, expression of the transcription factor RORC2 (*retinoic acid receptor-related orphan receptor 2*), and cell surface expression of CCR4, CCR6, CD161, and IL-23R (2–4). RORC2 is an isoform of RORγ (*nuclear receptor ROR-gamma*) encoded by the *RORC* gene, known as RORγt, in mice (5).

Different homologous genes encode the cytokine members of the IL-17 family (6). This family is composed of six members: IL-17A, IL-17B, IL-17C, IL-17D, IL-17E, and IL-17F (7). IL-17A is the most studied and is directly called IL-17 in many studies (6). Th17 cells also can produce other cytokines, such as IL-21 and IL-22, whereas cells producing IL-17 and IFNγ are referred to as Th1/Th17 cells (3). Th22 cells producing IL-22, IL-13, IL-26, and TNFα but not IL-17 and IFNγ were separated from the Th17 cell subset in 2009 (8).

Th17 cells are located primarily at barrier surfaces in the gastrointestinal tract, lung, and skin, participating in mucosal immunity (9). In the GALT (*Gut-Associated Lymphoid Tissue)*, mainly composed of Peyer’s patches, other lymphoid follicles, and lamina propria, Th17 cells represent 80 to 90% of the total CD4+ T cells (10). Th17 cells also represent a significant quantity of the T cells located in female genital mucosal tissues (11).

Extensive evidence has shown that Th17 CD4+ T cells are highly susceptible to HIV-1 (*human immunodeficiency virus*) infection and are rapidly depleted from gut mucosal sites (12, 13). Numerous mechanisms recently have been proposed to explain the susceptibility of Th17 cells to HIV infection. This review examines the current state of knowledge about: i) the definition and characteristics of conventional and unconventional Th17 CD4+ T cells; ii) data proving that Th17 CD4+ T cells are a preferential target for HIV reservoirs; and iii) cellular and molecular mechanisms contributing to HIV infection and HIV replication in Th17 CD4+ T cells.

### Conventional and Unconventional IL-17–Producing Cells

Conventional and unconventional CD4 T cells can produce IL-17. Conventional CD4 T lymphocytes are characterized by αβ TCR chains (1), whereas unconventional T cells harbor γδ TCR chains (14), semi invariant TCR (Vα7.2) on MAIT cells (*Mucosal-Associated Invariant T cells*), and invariant TCR (Va24-Ja18 paired with Vb11) on iNKT (*invariant Natural Killer cells*). Some studies have described γδ T cells as the most significant source of IL-17 secretion during infection (14, 15). MAIT cells, which are mainly CD8 T cells located in the GALT and peripheral organs, produce IL-17 once activated (16, 17). NKT cells (18) (*Natural Killer T cell*), and particularly iNKT cells (19, 20), also produce IL-17 upon appropriate stimulation.

### Th17 Profile’s Development

**Figure 1:**
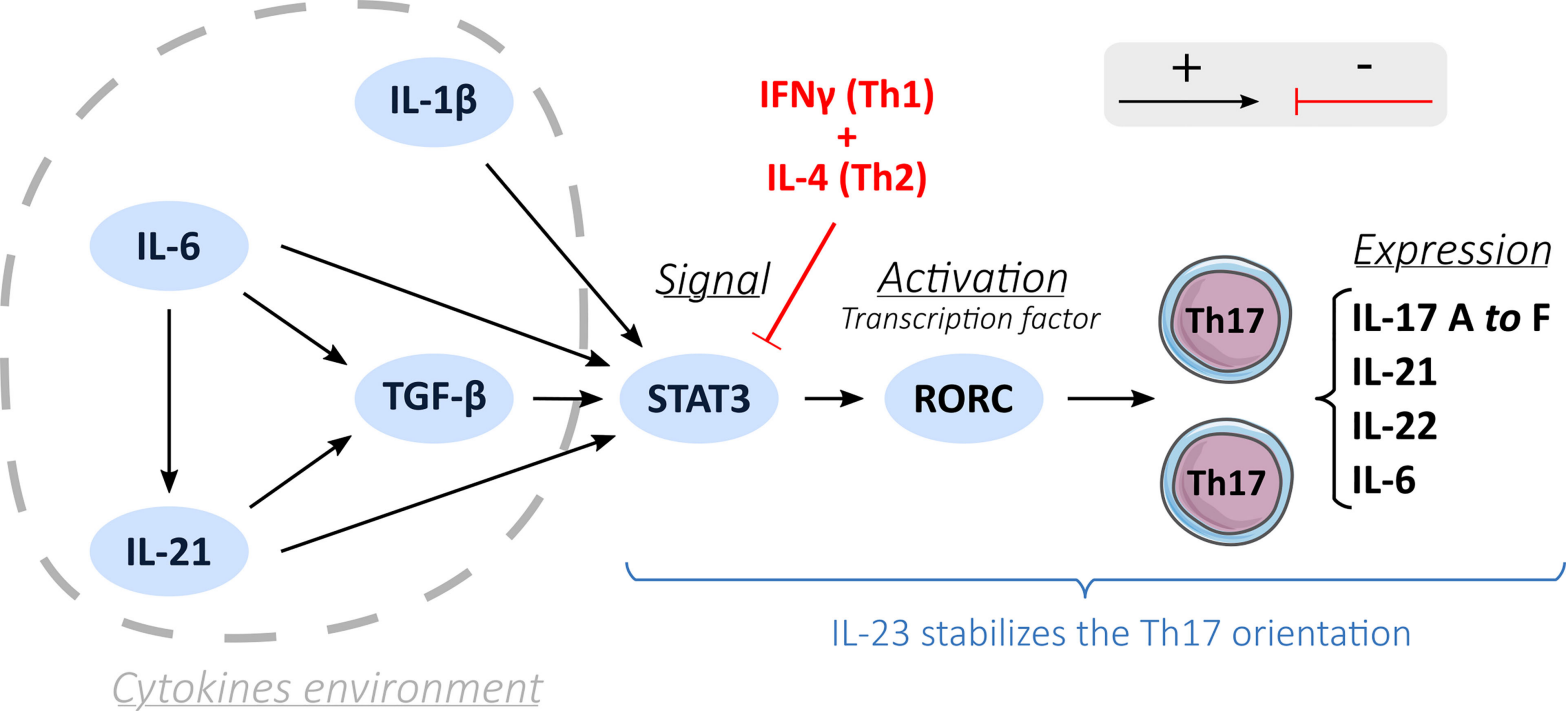
Th17 differentiation process. A description of the Th17 orientation process through different activation factors (black arrows): cytokines environment, induction by STAT3 and activation by a transcription factor RORC and the inhibition of the Th17 orientation process by the Th1 and Th2 cells (red arrows). IL-23 stabilizes the Th17 orientation.

In an appropriate cytokine environment and under co-stimulation, the recognition of a peptide antigen-class II major histocompatibility complex (MHC), which takes place through interaction with the TCR (T cells receptor), activates naïve T cells and can induce Th17 polarization (**Figure 1**) (21). Expression of the CD161 receptor by naive T cells may represent an initial condition for the fate of Th17 (4, 22). Among the factors involved in Th17 differentiation, TGF-β (23, 24) (*transforming growth factor beta*) and IL-6 (25) are essential. IL-6 induces the phosphorylation and dimerization of the transcription factor STAT3 (*signal transducer and activator of transcription 3*) (26). In turn, STAT3 induces the expression of the transcription factor RORγt and RORα, which are crucial for the orientation of Th17 by binding the IL-17A, IL-21, and IL-22 genes to the promoter regions (26). Activation by IL-6 requires support from TGF-β to induce the expression of RORγt (24), although the role of TGF-β has been questioned (25, 27). IL-1β may also be involved in the upregulation of RORγt (25).

It is thought that Th17 differentiation involves three pathways: i) the classical pathway involving IL-6 alongside TGF-β to induce the transcription of RORγt; ii) the alternative pathway, where IL-21 replaces IL-6; iii) and the third pathway involving IL-23 (24). TGF-β is also part of this third pathway by allowing the expression of the IL-23 receptor (IL-23R) on naïve CD4+ T lymphocytes (24). IL-23 then could engage and amplify the differentiation and growth of Th17 cells (28) and acts on their immune and proinflammatory functions by helping the recruitment of Th17 cells at the inflammation sites and increasing the production of IL-22 cytokines (29).

There is a crucial balance between proinflammatory Th17 and regulatory T cells (Treg) from a developmental perspective (13, 30). Th17 and Treg oriented CD4 T cells have interconnected development and antagonist functions (10, 31). The Foxp3 transcription factor allows the development of Treg cells and inhibits RORγt functions driving Th17 polarization (32). However, under certain immune circumstances, even a Treg cell may become Th17-like, producing IL-17 cytokines and expressing CCR6 at high levels, but a strong activation in the presence of IL-1β and IL-6 could stop this capacity (10).

### Immune Functions of Th17 Cells

**Figure 2:**
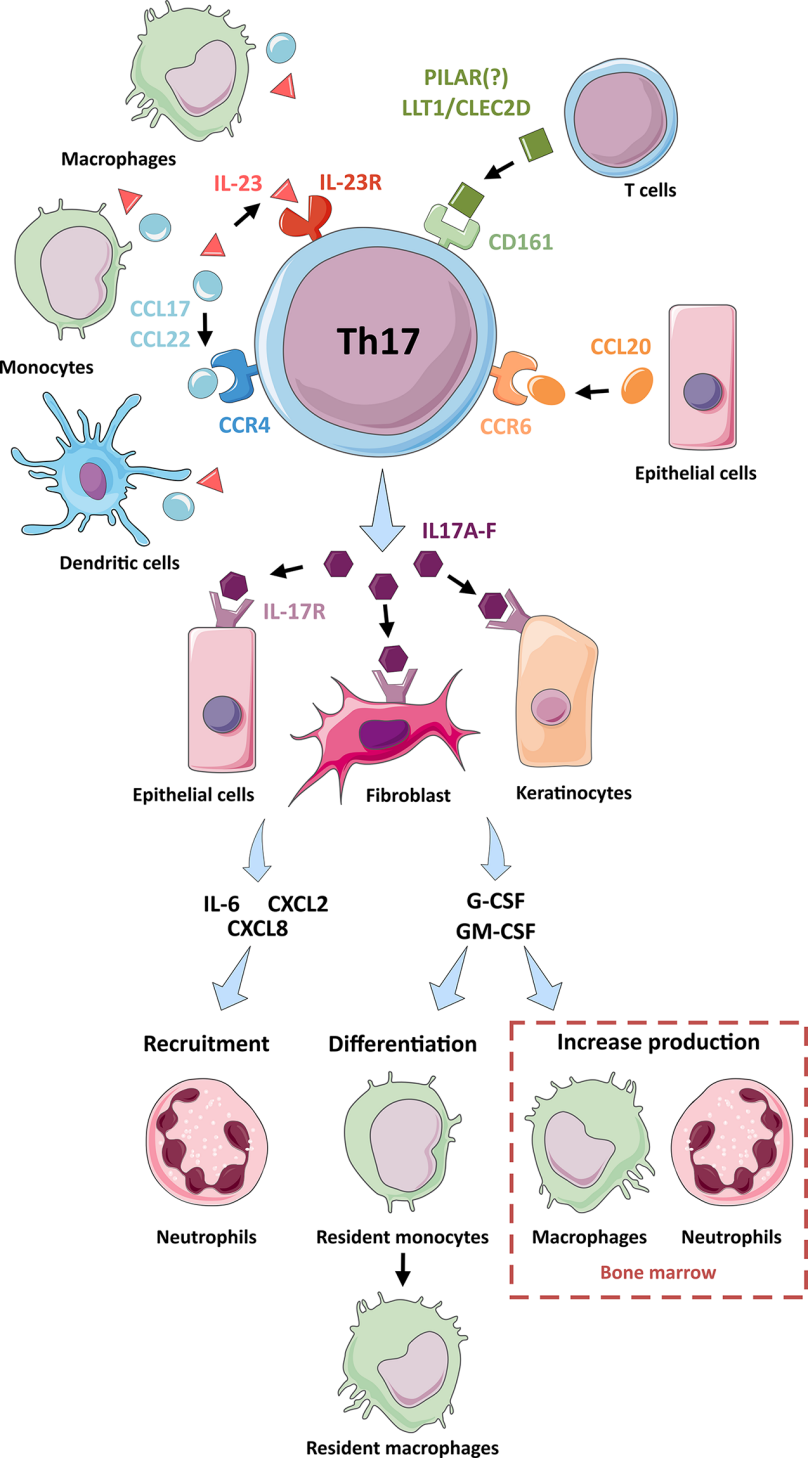
Interactions and functions of Th17 cells. A representation of the interactions of Th17 cells with other cell types: Through their production ligands for receptors on the surface of Th17 cells (IL-23R, CD161, CCR4 and CCR6), macrophages, monocytes and dendritic cells (CCL17, CCL22 and IL-23 production), T cells (LLT1, CLEC2D and PILAR) and epithelial cells (CCL20) participate in the activation of Th17 cells and their production of IL-17 cytokines. The IL-17 cytokines produced by Th17 cells activate epithelial cells, fibroblasts, and keratinocytes *via* their IL-17R surface receptor. Following this activation, these cells produce chemotactic factors (IL-6, CXCL2 and CXCL8) which participate in the recruitment of neutrophils. These cells also produce G-CSF and GM-CSF, which are involved in the differentiation of resident monocytes into resident macrophages and the increased production of macrophages and neutrophils in the bone marrow.

Th17 cells are abundant in mucosal-associated lymphoid tissues and play a vital role in the immune system’s defense against intestinal bacterial and fungi infections (33, 34). IL-23 produced by dendritic cells in contact with pathogens triggers the activation of mucosal resident memory Th17 cells (34) (**Figure 2**). The Th17 activated cells up-regulate IL-17 and IL-22 production (24, 30, 34), inducing mucosal epithelial cells to secrete antimicrobial peptides, such as members of the S100 family, HβD-2 (*human β-defensin 2*), and LCN2 (*lipocalin 2*) (34). The production of IL-17 also induces the expression of chemotactic factors, such as IL-6, CXCL2, and CXCL8, to recruit mainly neutrophils and macrophages to the infection site. IL-17 also activates the production of G-CSF (*granulocyte colony-stimulating factor*) and GM-CSF (*granulocyte-monocyte colony-stimulating factor*) by epithelial cells, fibroblasts, and keratinocytes (33, 34). These two growth factors allow the differentiation of mucosal resident monocytes into macrophages and increase the production and egress of macrophages and neutrophils from the bone marrow. The accumulation of neutrophils and macrophages in the mucosa with the activation of epithelial cells, endothelial cells, and fibroblasts promotes the secretion of proinflammatory cytokines (IL-1, IL-6, IL-23). These cytokines fuel the production of antimicrobial peptides and the local inflammatory response to attract other immune cells to the site of inflammation (34).

## Laboratory Methods for Identification of Th17-Oriented CD4 T Cells

In addition to identifying Th17 cells by their cytokine phenotype, cell surface phenotypic expression is used to define and study this cell subset (**Table 1** and **Figure 2**). The CCR6 chemokine receptor is the main surface marker of Th17 lymphocytes (2). However, CCR6 expression is not limited to Th17 cells since it also is present on B cells and dendritic cells located in lymphoid tissues of the intestinal mucosa. CCR6 is a receptor of the β-chemokine family (66). Unlike other chemokine receptors, CCR6 has CCL20 as the sole chemokine ligand. CCL20 is expressed at a low basal level, but it increases in the presence of proinflammatory signals. CCR6 also has a low affinity for two microbial peptides: ligands β-defensin 1 and β-defensin 2 (66). CCL20 signaling causes the chemotaxis of Th17 cells towards the gut and other barrier surfaces. In this pathway, this homing receptor is essential for the recruitment of Th17 lymphocytes (66). A study in 2016 reported four Th17 sub-populations: classical Th17 (CCR6+ CXCR3- CCR4+), Th1/Th17 (CCR6+ CXCR3+ CCR4-), double-negative (CCR6+ CXCR3- CCR4-) and double-positive (CCR6+ CXCR3+ CCR4+.) (60) These four sub-populations produce IL-17A but differ in their capacity to produce other cytokines such as IL-17F, IL-10, IL-13, IL-22, IFNγ, GM-CSF, and the CCR6+ double-negative population is described as a significant producer of IL-17A and IL-17F (60).

**Table 1:** Phenotypes used to characterize and sort Th17 cells.

Th17 cells identification methods:	References:
***IL-17+***	Harrington et al. (1)
	Annunziato et al. (3)
	Maek-A-Nantawat et al. (35)
	Brenchley et al. (36)
	Macal et al. (37)
	Ndhlovu et al. (38)
	Prendergast et al. (39)
	Chege et al. (40)
	Brandt et al. (41)
	McKinnon et al. (42)
	He et al. (43)
	Alvarez et al. (44)
	Kim et al. (13)
	McKinnon et al. (45)
	Li et al. (46)
	Saxena et al. (47)
	Caruso et al. (48)
	Caetano et al. (49)
***CCR6+ CCR4+ CXCR3-***	Acosta-Rodriguez et al. (2)
	Gosselin et al. (12)
	Becattini et al. (50)
	Ruffin et al. (51)
	Hani et al. (52)
	Pardons et al. (53)
***IL-17+ IFNγ+/-***	El Hed et al. (54)
***CD161+***	Maggi et al. (55)
	Li et al. (56)
***CCR6+ β7+*** *or* ***β7-***	Monteiro et al. (21)
***CCR6+ CXCR3- CD161+ IL-17+***	Cosmi et al. (4)
***CCR6+ CD161+ IL-23R+ CD26++***	Bengsch et al. (57)
***CCR6+ IL-17+***	Alvarez et al. (44)
***RORC2+ IL-17+***	Rodriguez-Garcia et al. (11)
***CCR6+ CCR4+***	Cleret-Buhot et al. (58)
***CCR4+ CCR6+ CXCR3- CD161+*** *(surface staining) vs.* ***IL-17+ IFNγ-*** *(intracellular staining)*	Dunay et al. (59)
***CCR6+CCR4+*** *(Th17)*	†Wacleche et al. (60)
***CCR6+CXCR3+*** *(Th1/Th17)*	
***CCR6+ CCR4- CXCR3-*** *(CCR6+ DN)*	
***CCR6+ CCR4+ CXCR3+*** *(CCR6+ DP)*	
***CCR6+***	Bolduc et al. (61)
	Gosselin et al. (62)
	Planas et al. (63)
***CCR6+ CXCR3-***	Côté et al. (64)
***CCR6+ CD161+ CXCR3-***	Zaunders et al. (65)

In this table, we find the different surface or intracellular markers used in the cited references to describe, study or sort Th17 cells.

† In 2016, Wacleche et al. described four populations of IL-17 productive cells: Th17, Th1/Th17, CCR6+ double-negative (DN), and CCR6+ double-positive (DP).

The CD161 surface receptor, formerly called NKR-P1 (*Natural Killer Receptor*), is the second hallmark of Th17 cells. CD161 can be expressed on αβ and γδ T cells, on the majority of NK cells, and NKT and MAIT cells (22). The physiological ligand of CD161 is LLT1 (*the lectin-like transcript-1*), belonging to the CLEC2 family (*C-type lectin domain 2 family*). This ligand could promote the transendothelial migration of Th17 effectors into tissues after recruitment *via* the CCL20-CCR6 interaction (22).

The first description of the CD161 receptor as a marker for Th17 cells was published in 2008 (67). In the CD161+ cell population, we can identify the Th17, Th1, and Th1/Th17 profiles, whereas in the CD161- population, we can only find the Th1 and Th1/Th17 profiles. The association of CCR6 and CD161 surface markers can allow, therefore, the identification of the vast majority of Th17-oriented cells.

IL23-R and CD26 can also be used to complete the cell surface phenotype of Th17 cells. IL-23R is a receptor expressed on the surface of Th17 cells (2, 3, 55). IL-23 is a heterodimer with a p40 subunit common to the cytokine IL-12 and a different p19 subunit, produced by monocytes, macrophages, and activated dendritic cells (68). On Th17 cells, the binding of IL-23 to its receptor IL-23R induces their activation (69). Hence, the cytokine IL-23 plays a role in the proinflammatory process mediated by Th17 cells. CD26 (*dipeptidyl-peptidase IV*) expression also can be used to identify Th17 oriented cells (57). CD26 is an active enzyme involved in the activation of T cells and found in tissue inflammatory responses. Th17 oriented cells have a high expression of CD26, which also is expressed to a lesser extent by Th1 and Th2 oriented cells.

## HIV Infection and Th17 Cells

### Susceptibility of Th17 Cells to HIV Infection

Mael-A-Nantawat’s team first demonstrated the susceptibility of Th17 cells to HIV infection in 2007 (35). Compared to Th1 and Th2 oriented CD4 T cells, Th17 cells show a superior permissivity and the highest level of HIV DNA integration *in vitro* and *in vivo* (12, 39). In contrast, during SIV (*simian immunodeficiency virus*) infection of rhesus macaques, Th17 cells are not preferentially infected compared to CD4+ memory T cells (70). These data suggest a limitation of using the SIV model to study differences in infectivity between Th17 cells and other CD4+ T cells. Th17 cells are enriched for different HIV dependency factors (HDFs) essential for virus replication (58, 71). The mechanisms allowing this enhanced permissibility can be separate into the entry and post-entry phases of HIV infection.

#### Entry level

**Figure 3:**
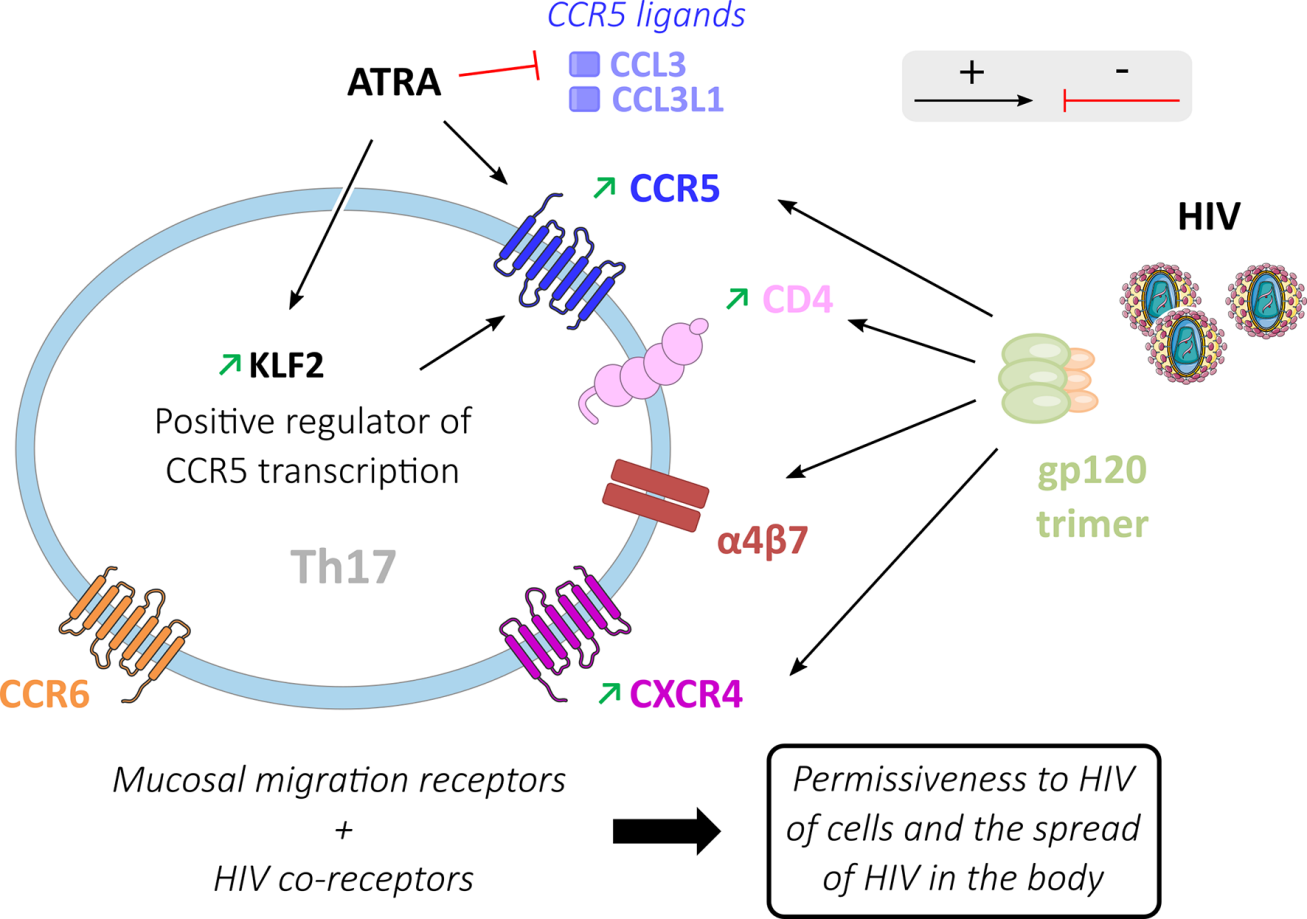
HIV entry process in Th17 cells. Activating (black arrows) or inhibiting (red arrows) factors are involved in HIV entry into Th17 cells. Mucosal migration receptors (α4β7 and CCR6) and HIV gp120 binding to co-receptors (CD4, CCR5, CXCR4 and α4β7) increase HIV permissiveness in these Th17 cells where these receptors are preferentially expressed. ATRA inhibits the production of classical ligands of the CCR5 receptor (CCL3, CCL3L1) thereby promoting the interaction of CCR5 with gp120. ATRA also increases the expression of KLF2 in Th17 cells, a positive regulator of CCR5 transcription.

HIV entry requires an attachment with the CD4 receptor, an interaction with either the CCR5 or CXCR4 co-receptors, and is facilitated by the presence of the α4β7 receptor (**Figure 3**). CD4+ helper T cells expressing these receptors are the primary target of HIV (11, 13, 44, 72). Whereas CD4 T cells express mainly CXCR4 and infrequently CXCR4 plus CCR5 co-receptors, CD4 Th17-oriented cells frequently express both CCR5 (3, 21, 54) and CXCR4 (3, 44). Consequently, Th17 cells are permissive *in vitro* to infection by viruses that have R5 (CCR5) or X4 (CXCR4) tropism (39, 56).

The CD4 and CXCR4 expression in Th17 oriented cells may be modulated by cell activation. Following *in vitro* polyclonal stimulation by anti-CD3/CD28, IL1-β, and IL-23, the subsets of CCR6+ CD4 T cells show higher expression of CD4 and CXCR4 than CCR6- cells, which may contribute to the greater permissiveness of Th17 cells to HIV infection (44). CCR5+ CD4+ memory T cells in healthy subjects are composed of 10 to 20% of Treg cells, 20 to 50% of Th1 cells, and 20 to 40% of Th17 cells (65). Different teams have shown that Th17 cells present a significantly higher expression of the CCR5 co-receptor compared to CCR6- cell subtypes (12, 56, 61, 63, 73). ATRA (*All-Trans Retinoic Acid*), a vitamin A metabolite produced by dendritic cells in the GALT (74), increases the expression of CCR5 in Th17 cells (21). ATRA induces a higher expression of CCR5 on memory CCR6 CD4 T cells co-stimulated by CD3/CD28, whereas its effect is modest on CCR6 negative cells (21). ATRA up-regulates transcription of KLF2 (Krüppel-like Factor 2) (63), a positive regulator of CCR5 transcription (75), and down-regulates the CCR5 ligands CCL3 and CCL3L1 (63), which have been shown to decrease the risk of HIV infection *in vivo* (76). Furthermore, ATRA plays a key role in the homing capacity of inflammatory Th17 cells in the intestine through a positive effect on CCR9 and α4β7 expression (77), which are required for cell migration to the gut. Hence, ATRA could be one of the factors involved in the permissiveness of Th17 cells to HIV infection by CCR5 expression and homing to the GALT.

The homing receptor α4β7 is strongly expressed on gut Th17 cells. The α4β7 receptor is an accessory binding target of the gp120 receptor (44, 63, 77, 78). Compared with the CD4 receptor, the α4β7 heterodimer is prominent because of its larger size. This characteristic makes α4β7 an efficient receptor for the capture of HIV particles (79). HIV attachment to the cell facilitates ensuing interaction with the CD4 entry receptor and the dissemination of the virus between cells (78). α4β7+ cells have been reported to be a preferential target of HIV during acute HIV-1 infection (80). Th17 cells are more permissive to HIV than CCR6- cells with low α4β7 expression (44). Th17 cell expression of mucosal migration receptors (CCR6 and α4β7), as well as HIV co-receptors (CD4, α4β7, CCR5, and CXCR4), participates in the remarkable capacity of these cells to disseminate HIV following initial infection (21).

#### Post-Entry Level and Replication

**Figure 4:**
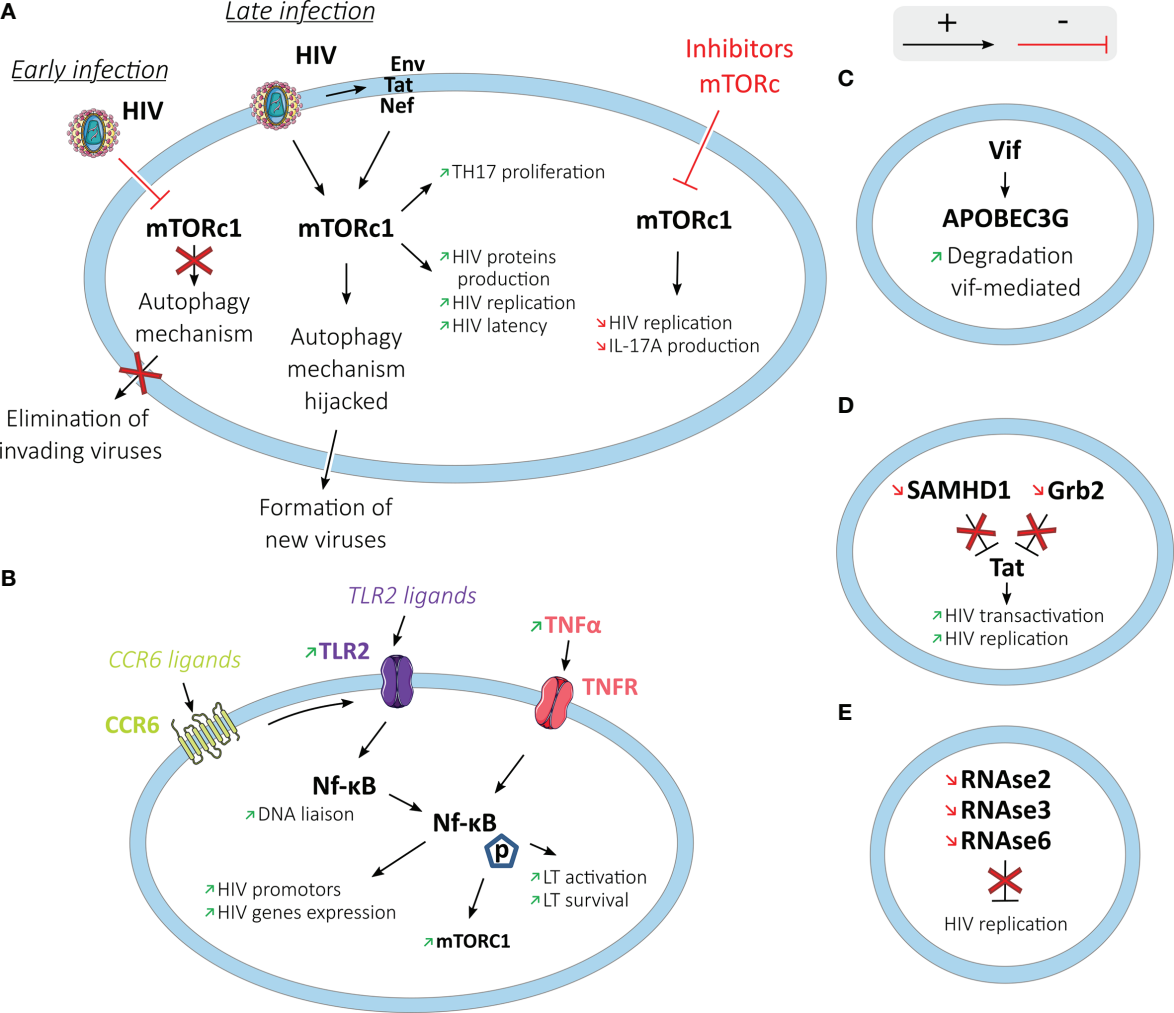
HIV post-entry process and replication in Th17 cells. Th17 cells and HIV have different interactions (activating in black arrows and inhibiting in red arrows) to promote HIV replication in these cells. **(A)** In the mTOR pathway: at an early stage of the infection, HIV stops the autophagy mechanism directed by mTORc1 to block the elimination of invading viruses. At a later stage, HIV interacts with mTORc1 to hijack the autophagy mechanism to help form new viruses. By interacting with HIV or its proteins (Env, Tat, or Nef), mTORc1 also increases Th17 proliferation, HIV protein production, HIV replication, and latency. **(B)** by interaction with NF-κB: increased production of TNFα and TLR2 ligation increase in Th17 cells NF-κB translocation and activity, leading to increased HIV replication. Increased NF-κB activity also increases the activity of the mTORc1 pathway and the activation and survival of T cells. **(C)** Th17 cells exhibit Vif-mediated degradation of the HIV restriction factor, APOBEC3G. **(D)** SAMHD1 and Grb2 are downregulated in Th17 cells, allowing increased HIV transactivation and replication. **(E)** RNase2, RNAse3, and RNAse6 genes known to inhibit viral replication are downregulated in Th17 cells.

After cell entry, the virus uses its enzymes and host machinery to reverse transcribe HIV RNA, integrate into the human genome, produce viral proteins, and form new viruses. Signaling pathways of Th17 cells such as mTORc1 (*mammalian Target Of Rapamycin Complex 1*) are involved in the post-entry steps of HIV replication (63) (**Figure 4**).

Activation of mTORc1 enhances Th17 differentiation, whereas mTORc1 dysfunction impairs it (81). In contrast, mTORc1 and mTORc2 inhibit Treg differentiation (82, 83). mTORc1 controls autophagy, which is an intracellular protective mechanism recycling cellular elements and eliminating newly formed viruses (84).

HIV-1 interacts with mTORc1, inhibiting or activating the mTORc1 complex (85). During the acute infection stage, different HIV-1 proteins inhibit mTORc1 activity, limiting autophagy. At a later stage of the infection in T cells, macrophages, dendritic and neuronal cells, HIV-1 hijacks the autophagy mechanism using the autophagosomal membrane to assemble viruses (86, 87). It has been shown that ATRA exposure increases mTOR expression and phosphorylation and HIV replication in Th17 cells (63). In contrast, metformin, which has an indirect inhibiting activity on mTOR, is shown to significantly decrease its activation through its phosphorylation in colonic CCR6+ cells (88). mTORc1 inhibitors such as Rapamycin and INK128 block HIV transcription through Tat-dependent and independent mechanisms (89). Tat is an HIV protein that acts as a viral transactivator.

Several HIV dependency factors (HDFs) were overexpressed in Th17 oriented CD4 T cells compared to Th1, Th2, and Th1/Th17 cells (58). Hence, higher expression of PAK2 (*p21 Activated Kinase 2*), PI3K (*Phosphoinositide 3-Kinase*), ZAP-70, and Lck (*lymphocyte-specific protein tyrosine kinase*) were observed using genome-wide transcriptional profiling (58). Binding of PAK2 by the HIV protein Nef impairs cell functions and facilitates virus replication by disrupting the dynamic rearrangements of the actin cytoskeleton in response to stimulation (90). PI3K participates in the negative control of MHC1 (*Major Histocompatibility Complex 1*) molecules mediated by the HIV protein Nef (91). ZAP-70 and Lck increase TCR signal and facilitate HIV replication by inducing cell activation upon weak TCR signals (92–94).

Some transcripts associated with a restriction of HIV replication are down-regulated in Th17 cells compared to Th1 cells, like Grb2 (*Growth factor Receptor-Bound protein 2*), known to inhibit HIV-1 LTR transactivation mediated by Tat during HIV replication (58, 95). Th17 cells present an enhanced Vif-mediated degradation of APOBEC3G (Apolipoprotein B mRNA editing enzyme, catalytic polypeptide-like 3G), an HIV restriction factor (73, 96). SAMHD1 (*SAM domain and HD domain-containing protein 1*) is also found to be down-regulated in Th17 cells (51). The reverse transcriptase activity requires the intracellular pool of dNTPs to replicate HIV; however, SAMHD1 can block this replication in dendritic cells, macrophages, monocytes, and CD4+ T cells by depleting the intracellular pool of dNTPs (97, 98). SAMHD1low circulating memory CD4+ T cells are enriched in Th17 cells (median at 41%) and demonstrate high levels of HIV-1 DNA (52). Higher levels of HIV-1 DNA were observed in the CD45RO+ SAMHD1low memory population (4.5 HIV-1 DNA log copies/10^6^cells) compared to CD45RO+ SAMHD1+ memory cells (3.8 HIV-1 DNA log copies/10^6^cells, p=0.009) and CD45RO- SAMHD1+ naive cells (3.1 HIV-1 DNA log copies/10^6^cells, p<0.0001). Likewise, a higher population of p24 producing cells was detected within the SAMHD1low memory population in viremic and treated individuals (52). SAMHD1low memory cells harbored a higher percentage of Ki67 expression (15.2%) compared to SAMHD1+ memory cells (3.37%) and SAMHD1+ naïve cells (0.23%). A positive correlation was observed between HIV-1 DNA levels and Ki67 expression.

NF-κB is another transcription factor involved in T cells activation and cell survival (99). NF-κB also can initiate HIV genome transcription by liaison with the HIV LTR enhancer region promotor, which contains two NF-κB binding sites (100). Th17 cells have been found to have an increased nuclear translocation activity of NF-κB (*nuclear factor-kappa B*) and DNA liaison compared to Th1 cells (58). The increased activity of NF-κB in Th17 cells compared to Th1 cells is associated with higher production of TNF-α (63). TNF-α (*Tumor Necrosis Factor-α*) can increase HIV replication by activating NF-κB (101). Once activated, TLR2 (*Toll-like receptor 2*) leads to NF-κB translocation (102). TLR2 is a member of the TLR family, which can recognize different PAMPs (*Pathogen-associated molecular patterns)*, and thus different types of pathogens (103). TLR2 activity could enhance the susceptibility of CD4+ T cells to HIV-1 productive infection, notably by significantly increasing p65 phosphorylation (NF-κB activity marker) in Th17 cells (61, 104). Th17 cells, quiescent or activated, present higher levels of TLR2 expression in FACS analysis (61). Following *in vitro* activation, TLR2 ligation induces a higher level of HIV-1 replication in Th17 cells compared to CCR6- cells (61).

Increased Lck and ZAP-70 expressions in Th17 cells compared to Th1 cells can enhance the capacity of Th17 cells to be activated in response to weak TCR signals (58). This enhanced capacity to increase cell metabolism creates an environment suitable for HIV replication. RORγt, the key transcription factor for Th17 cell orientation, can promote HIV-1 replication (105). RORγt is thought to regulate viral gene expression through its binding to the HIV-1 LTR. Th17 cells can also increase intracellular HIV replication by a lower expression of some members of the RNase A (*Ribonuclease A*) superfamily previously described as factors inhibiting viral replication (60, 73). RNase2, RNase3, and RNase6 genes, all known to have viral replication inhibition activity, are expressed at lower levels in Th17 cells compared to CCR6- cells on RNA microarray analysis (73).

Overall, the up-regulation of numerous factors facilitating HIV replication, such as PAK2, ZAP-70, Lck, NF-κB with TNF-α or TLR2, alongside the down-regulation of factors inhibiting HIV, such as Grb2 and SAMHD1, in Th17 oriented CD4 helps explain why these cells represent a favored target for HIV.

### Quantitative Depletion to Th17 Cells and Effects of Antiretroviral Therapy

#### Depletion of Th17 Cell Pool

The severe depletion of Th17 polarized CD4 T cells is observed early during HIV infection (73). Many studies have shown that the depletion of Th17 cells occurs in the blood compartment (13, 36, 40, 54), the intestinal mucosa (13, 36, 40, 64), and the genital mucosa (42, 45). Furthermore, the number of Th17 cells circulating is inversely correlated with HIV viral load (59).

#### Impact of ART in Th17 Cell Recovery

ART reduces plasma HIV to undetectable levels and induces a progressive replenishment of CD4+ T cells (38). However, after four years of effective therapy, ART appears to be only partially effective in restoring Th17 CD4 T cells (40). After six years on ART, the Th17 cell frequency can be normalized in blood, but recovery of Th17 function is highly variable between individuals (48). Early versus delayed initiation of ART also affects Th17 cell recovery. The number of Th17 cells in the intestinal mucosa could return to a normal level when treatment is initiated in the acute infection phase but not when initiated during the chronic phase of HIV infection (13, 40, 44).

Th17 CD4 T cell recovery also appears variable in the different anatomical compartments and between the different Th17 cell subsets (40). The pool of mucosal Th17 cells is less rapidly restored than the blood counterpart (40, 106). A study of Th17 CD4 T cells in female genital mucosa has observed partial recovery of Th17 cells under ART with a low capacity of Th17 related cytokine production (48). The difficulty encountered by Th17 cells in restoring their cell pool may be partially explained by the decrease in CD4+ CD161+ cells, considered Th17 cell precursors with mucosal homing capacity (22, 67). In untreated subjects with a chronic HIV infection, the proportion of circulating CD4+ CD161+ cells is lower than in healthy controls (39).

The CCR6+ CXCR3- CCR4- (double negative) cell subtype may be preserved during HIV infection in blood and lymph nodes, unlike the Th17 (CCR6+ CXCR3- CCR4+), Th1/Th17 (CCR6+ CXCR3+ CCR4-), and double-positive (CCR6+ CXCR3+ CCR4+) subtypes, which are more severely depleted and only partially restored under ART (60). CCR6+ double negative cells may be oriented to the three other subtypes based on the differentiation signals emitted (60).

CD4+ T cells have been explored by transcriptional analysis in HIV-infected individuals maintaining an undetectable HIV-1 viral load over time in the absence of ART (elite controllers) (107). Genes associated with Th1, Th17, and Th22 orientation were highly expressed in HIV-specific CD4+ T cells in elite controllers compared to untreated patients with a progressive HIV infection, in particular the genes enabling the production of the transcription factor RORC driving Th17 differentiation and the cytokines IL-17 and IL-22 (107). A high production of IL-17A, IL-17F, and IL-22 is therefore found in elite controllers. In contrast, in untreated subjects with a progressive disease, the production of these cytokines is low or undetectable. After initiation of ART, the difference persists between the elites controllers and subjects with a chronic infection, with a low expression in untreated subjects of genes associated with Th17 cells amongst HIV-specific CD4+ T cells (107).

### Functional Impairments Related to Th17 Cell Depletion

The impaired functionality of the Th17 cell population on ART may contribute to functional problems observed in people living with HIV.

#### Intestinal Barrier Function

Th17 cells play a crucial role in regulating gut mucosal immune defense against microbial pathogens (108). Depletion of CD4+ T cells in gut-associated lymphoid tissues occurs early during HIV infection and persists durably on ART, disrupting the intestinal barrier’s integrity.

The early decrease of mucosal Th17 cells from the acute HIV infection, and incomplete replenishment of Th17 cells on ART, lead to a frequent and prolonged impairment of the antimicrobial immune defense (54). Plasma levels of LPS (*lipopolysaccharide*) and soluble CD14 levels, markers of microbial translocation, are increased in untreated and treated HIV-infected subjects (40). Plasma levels of BDG ((1➔3)-β-D-Glucan), a compound of the fungi membrane and a marker of fungal translocation, are also elevated in HIV-infected individuals (109). These bacterial and fungal translocations are associated with systemic immune activation and tissue inflammation (109). Cells in inflamed gut tissue are described as having a 27-fold higher frequency of HIV DNA than cells in non-inflamed gut tissue (110). This immune activation and inflammation is thought to lead to an increase in the frequency of other conditions such as metabolic and cardiovascular diseases (111).

In turn, bacterial translocation and chronic immune activation impair the cytokine expression of Th17 cells. Th17-oriented cells naturally produce IL-10 in minimal quantities and proinflammatory cytokines (IL-17, IL-22, TNFα, and IFNγ) in large quantities (13). In uninfected controls, a median of 69.7% of sigmoid Th17 cells produce TNFα, and 0.3% produce IL-10 (13). In the early stages of an HIV infection, IL-10 production increases in sigmoid Th17 cells (3.5%), whereas TNFα production decreases (43.4%), allowing an IL-10/TNFα imbalance (13). This ratio in IL-10/TNFα production normalizes in the chronic stage of HIV infection and under long-term ART, with increased production of TNFα in sigmoid colon Th17 cells (88.0%) in ART subjects (versus 69.7% in uninfected controls at p=0.002).

#### The Dynamic of Th17/Treg Balance During HIV Infection and on ART

The relative proportion of Th17 versus Treg cells is essential to maintain immune homeostasis under physiological conditions. In healthy controls, the Th17/Treg cells ratio is usually between 1.0 and 1.2. HIV infection disrupts the Th17/Treg ratio axis (10) with a balance shifted in favor of Treg cells. A rapid decrease of Th17 cells is observed in ART naïve subjects, while the decline in the number of Treg cells is gradual (39). Both the number and frequency of Th17 cells decrease during chronic HIV infection, whereas Treg cell numbers decrease slowly and the Treg proportion increases. The Th17/Treg ratio decay becomes severe at a late stage of HIV infection, with values ranging from 0.75 to 0.2. In contrast, data suggest that Th17/Treg balance may be preserved in long-term non-progressors and maintained at an average level in elite controllers (47, 49). Tryptophan catabolism into immunosuppressive kynurenine (Kyn) by indoleamine 2,3-dioxygenase (IDO) could contribute to this difference in the Th17/Treg ratio (112). The enzyme IDO, which catabolizes Trp to Kyn, is also described as a marker of inflammation for the study of HIV-1 progression (112). IDO is thought to impact the Th17/Treg balance by stimulating Treg cells and blocking their reprogramming into Th17 (112). Early administration of ART could block this Treg cell stimulation and restore the Kyn/Trp ratio (113).

Monitoring Treg cells in HIV-infected patients beginning ART shows a rise in the frequency of Treg cells circulating in the first weeks and a subsequent decrease to reach values similar to healthy controls (114). While Treg recovery is rapid during the first months of ART, the gain of Th17 oriented cells is delayed, contributing to the imbalance of the Th17/Treg ratio (43). Th17/Treg balance can be restored when treatment is initiated in the early phase of the infection and after prolonged ART associated with a gradual rise of Th17 cell numbers (43).

### HIV Reservoirs in Th17 Cells

One of the main characteristics of an HIV reservoir is its persistence under viral suppressive treatment (115). Different cell subsets located in different anatomical compartments comprise the pool of HIV-infected cells. Mucosal tissues such as the intestinal mucosa are enriched sites for HIV infection, replication, and reservoir (48, 64, 115, 116). HIV infection impairs intestinal mucosal immunity from the early phase of infection *via* a profound depletion of mucosal CD4 + T cells (117). The persistence of HIV reservoirs in the GALT in patients undergoing ART is a major obstacle to the eradication of HIV (118). GALT lymphocytes comprise a high proportion of effector memory and activated CD4+ lymphocytes (115). Hence, it is of prime importance to study Th17 cells for their ability to serve as an HIV reservoir (72).

#### Quantity and Heterogeneity of HIV Reservoirs in Th17-Oriented Cells

HIV persists in circulating and gut located CD4+ CCR6+ T cells in patients under treatment (62). In patients on ART, the CCR6+ population has significantly higher levels of HIV DNA in the blood (1.5-fold) and colon (2.8-fold) than CCR6- cells. CCR6+ cells also have a higher expression of the activation marker HLA-DR than CCR6- cells in the colon (62). The size of the HIV reservoir has been compared in CD161+ and CD161- cells from patients on ART (56). The quantity of HIV-1 DNA measured by qPCR was found to be 6.7-fold higher in CD161+ cells than in CD161- cells (56). The level of intact HIV reservoirs measured by Intact Proviral DNA Assay (IPDA) was 13.0-fold higher in the CD161+ cells than in the CD161 negative counterpart (56). Likewise, the number of replication-competent latent HIV measured by quantitative viral outgrowth assay (QVOA) was 2.1-fold higher in CD161+ cells than CD161- cells (56).

The different subsets of Th17 cells, classical Th17 (CCR6+ CXCR3- CCR4+), Th1/Th17 (CCR6+ CXCR3+ CCR4-), double negative (CCR6+ CXCR3- CCR4-) and double positive (CCR6+ CXCR3+ CCR4+), harbor HIV reservoirs. These different subsets of IL-17 producing cells contain integrated HIV DNA and are replication-competent HIV-infected cells (60).

Th17 cells can produce viral particles after four days of CD3/CD28 stimulation (60). Using a modified viral outgrowth assay, the authors have shown expression of HIV-p24 associated alongside IL-17A (60). *In vitro* infection of CD4 T cells shows that Th17 oriented cells account for 18% of the total population of infected cells and constitute a fifth of p24+ cells, whereas they represent only 6.2% of CD4 T cells (73). This observation has been confirmed *in vivo* using cytometry and single-cell analysis*;* among HIV-infected cells, Th17 cells (CCR6+ CXCR3- CCR4+) accounted for a quarter of p24-producing cells (53). Th17 cells presented higher p24 secretion in cell supernatants and a higher level of HIV RNA in sorted cells (73).

PCR quantification of HIV-1 DNA from patients under prolonged ART confirmed that Th17 and Th1/Th17 cells significantly contribute to the HIV reservoir relative to their frequency among T CD4 memory cells (119). In comparison, the Th1-oriented cells appeared to have a proportionate contribution to the HIV reservoir relative to their frequency, while Th2 cells appeared to have a minor contribution to the HIV reservoir. In this study, the follow-up of patients showed that the contribution of Th17 and Th1/Th17 cells to the HIV reservoir increased from 29% on short-term ART to 53% under long-term therapy, whereas it slightly lowered in Th1 cells (119).

#### Longevity, Proliferation Capacity, and Clonal Expansion

A long lifespan is an essential characteristic of the cells participating in the latent reservoir. Furthermore, the proliferation of latently infected cells generates clones of CD4 T cells that carry integrated proviruses (120).

In 2011, a team described Th17 cells sharing the characteristics of longevity and plasticity of stem cells (121). The Transcription Factor 7 protein (Tcf7) plays a crucial role in regulating self-renewal capacity *versus* differentiation of stem cells through its association with beta-catenin that operates in the absence of autocrine Wnt signaling (122, 123). *In vitro* Th17 cells have a higher expression of *Tcf7* associated with a significant accumulation of the protein β-catenin compared to Th1 cells and naive cells (121). By testing apoptosis resistance and survival capacity *in vitro*, Th17 cells showed less activation-induced cell death (AICD) and a remarkable ability to persist the pool of memory cells compared to Th1 cells (121).

The subset of CCR6+ double negative (CXCR3- CCR4-) cells regarded as CD4 T cells engaged toward the early development of Th17 orientation presents a transcriptional signature of self-renewal (60). Hence, some pathways and markers of human stem cells, such as NANOG, LEF1, and MYC, were found up-regulated in CCR6+ double negative cells, as well as TORC, an anti-senescence marker (60).

The link between long-term survival, proliferation capacities, and clonal expansion of the latent HIV-1 reservoir also was studied in CD161+ Th17-oriented cells and compared to CD161- cells (56). CD161+ cells presented a significantly higher expression of c-kit and Bcl-2, two critical molecules for cell survival (56, 124, 125). CD161+ cells also showed a higher expression OX40 (56), a protein associated with long-term survival and clonal proliferation of CD4+ cells (126). In patients on ART, clonal HIV-1 sequences were more frequently detected in OX40 positive CD4 T cells (127).

## Conclusion

In many respects, Th17 cells are a critical cell compartment of the HIV reservoir. Through their prominent location in the gastrointestinal tract and their participation in the mucosal tissues’ immunity, Th17 cells are actively implicated in a preferential anatomical site and cellular subset for HIV infection. Th17 cells participate in the dissemination of HIV following infection through their expression of mucosal migration receptors (CCR6, α4β7) and HIV co-receptors (CD4, α4β7, CCR5, and CXCR4) through different biological pathways. Th17 cells facilitate HIV replication at the entry-level as well as the post-entry and productive levels. The long-term cell survival and proliferation capacities of Th17 cells are characteristics that contribute to the persistence and clonal expansion of HIV in ART-treated persons. Hence, Th17 cells account for a large proportion of HIV reservoirs compared to their relative frequency among CD4 memory T cells. Innovative therapeutic approaches aiming to achieve an HIV cure should consider and take advantage of the characteristics and particularities of Th17 cells (128).

## Author Contributions

Writing, original draft preparation, figure creation, CR. Writing, review and editing, NV, FM, A-SB, JPR, PP, JR, and ET. All authors contributed to the article and approved the submitted version.

## Funding

CR was supported by the University of Montpellier, the Centre Hospitalier Universitaire (CHU) de Montpellier, Fédération Hospitalo-Universitaire Infections Chroniques (InCH).

## Conflict of Interest

The authors declare that the research was conducted in the absence of any commercial or financial relationships that could be construed as a potential conflict of interest.

## Publisher’s Note

All claims expressed in this article are solely those of the authors and do not necessarily represent those of their affiliated organizations, or those of the publisher, the editors and the reviewers. Any product that may be evaluated in this article, or claim that may be made by its manufacturer, is not guaranteed or endorsed by the publisher.
